# The effect of metyrosine/prednisolone combination to oophorectomy-induced osteoporosis

**Published:** 2012-07

**Authors:** Suleyman Salman, Serkan Kumbasar, Ahmet Hacimuftuoglu, Berna Ozturk, Bedri Seven, Beyzagul Polat, Cemal Gundogdu, Elif Demirci, Kadir Yildirim, Fatih Akcay, Turan Uslu, Ferrah Tuncel Daloglu, Halis Suleyman

**Affiliations:** 1 *Department of Obstetrics and Gynecology, Igdir National Hospital, Igdir, Turkey.*; 2 *Department of Pharmacology, Ataturk University, Medical Faculty, Erzurum, Turkey.*; 3 *Department of Nuclear Medicine, Ataturk University, Medical Faculty, Erzurum, Turkey.*; 4 *Department of Pathology, Ataturk University, Medical Faculty, Erzurum, Turkey.*; 5 *Department of Physical Medicine and Rehabilitation, Ataturk University, Medical Faculty, Erzurum, Turkey.*; 6 *Department of Biochemistry, Ataturk University, Medical Faculty, Erzurum, Turkey.*; 7 *Department of Physical Medicine and Rehabilitation, Fatih Sultan Mehmet Education and Research Hospital, Istanbul, Turkey.*

**Keywords:** *Osteoporosis*, *Metyrosine*, *Methylprednisolon*, *Alendronate*, *Ovariectomy*

## Abstract

**Background:** Osteoporosis is a chronic disease characterized by a decrease in bone mineral density (BMD) and corruption of the microarchitectural structure of bone tissue.

**Objective:** It was investigated whether methylprednisolone had a favorable effect on osteoporotic bone tissue in Oophorectomy induced osteoporotic rats whose endogenous adrenaline levels are suppressed with metyrosine.

**Materials and Methods:** Bone Mineral Density, number of osteoblast-osteoclast, bone osteocalcin levels and alkaline phosphatase (ALP) measurements were performed. Obtained results were compared with that of alendronate.

**Results:** Oophorectomy induced osteoporosis was exacerbated by methylprednisolone. Alentronate prevented ovariectomised induced osteoporosis, but it couldn’t prevent methylprednisolone +ovariectomised induced osteoporosis in rats.

**Conclusion:** Combined treatment with methylprednisolon and metyrosine was the best treatment for preventing osteoporosis but metyrosine alone couldn’t prevent osteoporosis in ovariectomised rats.

## Introduction

Osteoporosis is a chronic disease characterized by a decrease in bone mineral density (BMD) and corruption of the microarchitectural structure of bone tissue (1). 

Although numerous drugs and methods are used to treat osteoporosis, 210 million people are affected by osteoporosis worldwide and, in the U.S., 1.5 million women suffer from bone fractures caused by osteoporosis annually, demonstrating that current osteoporosis treatment is insufficient. Hence, there is a need for the production of better quality drugs and the development of efficient methods of osteoporosis treatment. Osteoporosis is usually considered a women’s disease, but it is also a serious health problem for men. As men have higher muscle and bone masses and do not experience menopause, osteoporosis is less frequent in men (2). 

The main cause of postmenopausal osteoporosis is lack of estrogen (3); decreased serum estrogen levels are associated with decreased BMD and increased risk of bone fractures. Estrogen has a protective effect on bones by decreasing the remodeling of bones, elongating the life of osteoblasts-which are responsible for bone production-and suppressing the maturation of osteoclasts (4, 5). 

Corticosteroid usage is one of the numerous contributing factors to osteoporosis development (6). The effects of glucocorticoids on bone are a decrease in bone production, increase in bone destruction, and decrease in BMD (7). Therefore, glucocorticoid use results in osteoporosis-and osteoporosis-related fractures (8). It has been shown that osteoporosis development is caused by changes in the balance of osteoblasts and osteoclasts, causing an increase in osteoclasts (9). Prednisolone, a glucocorticoid, was shown to induce serious adverse effects such as bone loss and bone fractures, even at doses as low as 7.5 mg/day (10). 

However, a study by Takeuchi *et al* demonstrated that the toxicity of glucocorticoids on tissues is converted to a positive effect in adrenalectomized rats (11). In a previous study, we showed that prednisolone completely prevents indomethacin-induced stomach ulcers in rats whose adrenalin levels are decreased (12). These records indicate that the negative effects of glucocorticoids on tissues may turn into positive effects when endogenous adrenalin is suppressed. The aim of our study is to investigate the effects of methylprednisolone, a glucocorticoid, in rats with ovariectomy-induced osteoporosis whose endogenous adrenalin levels are suppressed with metyrosine. 

## Materials and methods

**Animals**

Seventy female albino Wistar rats weighing between 210 and 220 gr were used in the experiments. Animals were provided by Atatürk University Medicinal and Experimental Application and Research Center and were kept and fed in groups under normal laboratory conditions before the experiments. 

In this study the animals were employed in accordance with the national guidelines for the use and care of laboratory animals and were approved by the local animal care committee of Ataturk University. Chemicals: Metyrosine (Demser, Aton Pharma Inc.), depomedrol (Eczacıbasi İlac San.), alendronate (fosamax, Merck-Sharp and Dohme), and thiopental sodium (pentotal-Abbott) were provided. 

**Experimental procedures**

70 rats were starved for 12 hours at first stage and then they were ovariectomized under 25 mg/kg thiopental sodium anesthesia (13, 14). The first ovariectomized rat group was administered 20 mg/kg metyrosine orally and the second group received 2 mg/kg alendronate orally by gavage. The third group of ovariectomized rats was given a combination of metyrosine (20 mg/kg oral) and methylprednisolone (10 mg/kg im). The fourth group of ovariectomized rats was given a combination of alendronate (2 mg/kg oral) and methylprednisolone (10 mg/kg im), while the fifth group received 10 mg/kg methylprednisolone (im). 

The sixth group of ovariectomized rats received distilled water and was used as the control. A seventh group of intact rats received distilled water was also used as a control. For three months, metyrosine was administered twice a day and alendronate was administered once a day; methylprednisolone was injected once every ten days for three months. At the end of three months, animals were sacrificed by a high dose anesthesia and their hipbones were removed and sent to Nuclear Medicine and Pathology departments for BMD, osteoblast, and osteoclast estimations. Additionally, blood samples taken from the hearts of the animals were sent to a biochemistry lab for osteocalcin and bone alkaline phosphatase assay. 

**Radiological investigation**

**Dual Energy X-RAY Absorptiometry (DEXA) estimations**

The femur bones of rats were evaluated in vitro after being surgically removed. Bone mineral density (BMD g/cm^2^) and bone mineral content (BMC g) were determined by the DEXA method using a Hologic QDR 4500 (Hologic Inc., Waltham, MA, USA) machine. Each measurement was performed by the same researcher and all analyses were done using the same region of interest (ROI) window size. 

**Pathological investigation**

Seventy operation materials (hipbones) were fixed in 10% buffered formalin solution for 48 h. Later the materials were decalcified in 5% nitric acid for 48 hours. After fixation and decalcification, three tissue samples from each rat were taken for routine follow-up procedure and these samples were put in paraffin blocks. After the follow-up procedure, 5 µm wide sections were taken from paraffin blocks for histopathological examination. After deparaffinization and rehydration, sections were stained with hematoxylin and eosin stain. Stained sections were examined under an Olympus BX5 microscope at 40x magnification by two independent pathologists. Each region of the sections was evaluated and the osteoblasts and osteoclasts numbers were recorded.

**Biochemical investigation**

Venous blood samples were collected into tubes without anticoagulant. Serum was separated by centrifugation after clotting and stored at 80^o^C until assayed. ALP activity was determined in Cobas 6000 (Roche) photometrical system with colorimetric method. Osteocalcin and Vitamin D3 (25-OH) levels were determined in E-170 (Roche) ECL system with electrochemiluminessence methot. Colorimetric assay in accordance with a standardized method ALP; (p-nitrophenyl phosphate and H_2_O ALP/Mg+ phosphate and p-nitrophenol) In the presence of magnesium and zinc ions, p nitrophenyl phosphate is hydrolyzed by phosphatases to form phosphate and p-nitrophenol. The p-nitrophenol released is proportional to the ALP activity and can be measured photometrically.

**Statistical analysis**

All results were shown as the means±standard error of mean (SEM). One-way analysis of the variance was used to evaluate the results. A value of p<0.05 was considered significant.

## Results

**DEXA results**

BMD was 0.137±0.03, 0.14±0.04 (p>0.05), and 0.143±0.01 (p<0.05) g/cm^2^ in control, metyrosine administered, and alendronate administered ovariectomized rat groups, respectively. The methylprednisolone administered ovariectomized rat group had a BMD of 0.126±0.02 (p<0.05) g/cm^2^, while the metyrosine and methylprednisolone and alendronate and metyrosine administered ovariectomized rats had BMD values of 0.158±0.02 (p<0.001) and 0.132±0.03 (p>0.05) g/cm^2^, respectively. Intact rats group had a BMD value of 0.159±0.05 (p<0.0001) g/cm^2 ^(Table I). 

**Pathological results**

**Figure 1:**
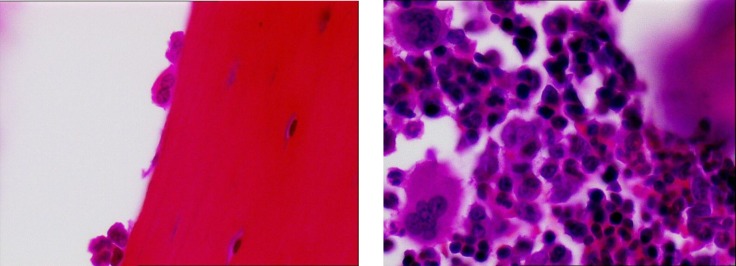
Osteoblasts (left) and osteoclasts (right) in ovariectomized control group

**Figure 2:**
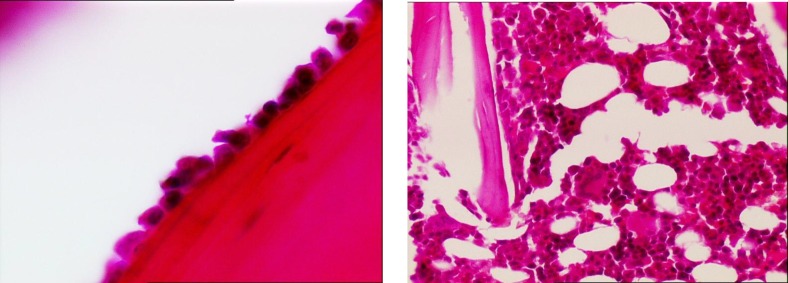
Osteoblasts (left) and osteoclasts (right) in intact control group

**Figure 3:**
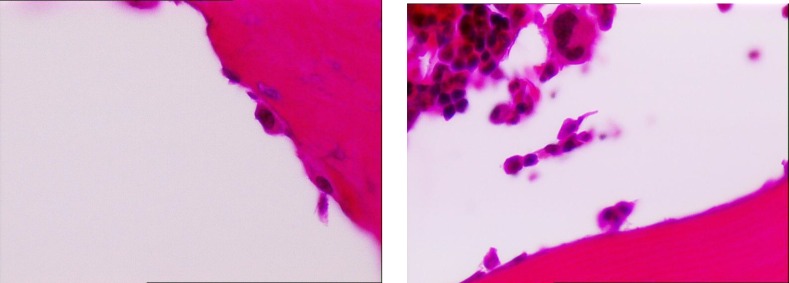
Osteoblasts (left) and osteoclasts (right) in metirosine administered ovariectomized rat group

**Figure 4:**
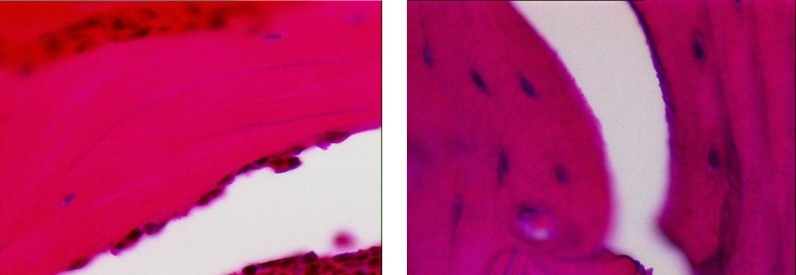
Osteoblasts (left) and osteoclasts (right) in alendronate administered ovariectomized rat group

**Figure 5:**
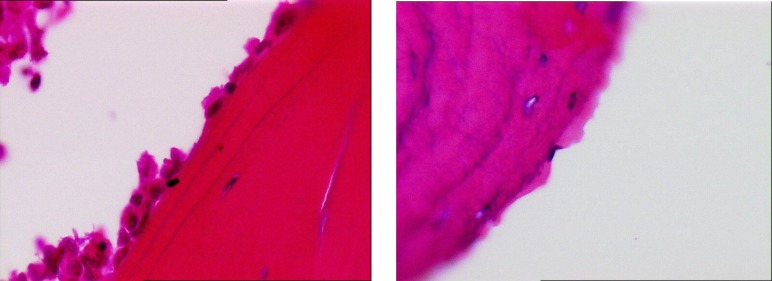
Osteoblasts (left) and osteoclasts (right) in metirosine + methylprednisolone administered ovariectomized rat group

**Figure 6:**
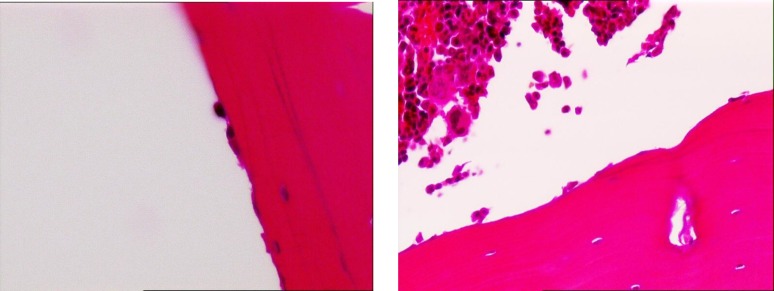
Osteoblasts (left) and osteoclasts (right) in alendronate + methylprednisolone administered ovariectomized rat group

The average number of osteoblasts and osteoclasts in the hipbones of oophorectomy group of rats were 3.5 and 3, respectively (Figure 1). The intact control group had an average of 14 osteoblasts and 2 osteoclasts (Figure 2). The metyrosine administered ovariectomized group had an average of 5 osteoblasts and 1 osteoclast (Figure 3). Alendronate administered ovariectomized rats had an average of 10 osteoblasts and no osteoclasts were found (Figure 4). Metyrosine and methylprednisolone combination administered ovariectomized rats had an average of 8 osteoblasts and no osteoclasts were found (Figure 5). Alendronate and methylprednisolone administered ovariectomized rats had an average of 2 osteoblasts and no osteoclasts (Figure 6). 

**Biochemical results**

**Osteocalcin levels**

The average osteocalcin level in the ovariectomized control rat group was 20±5.3; in the intact rat group it was 12.1±2.7 (p<0.001). Metyrosine and alendronate administered ovariectomized rats had average osteocalcin levels of 17.1±3.5 (p>0.05) and 14.3±2.7 (p<0.01), respectively. Average osteocalcin levels in metyrosine and alendronate and metyrosine and methylprednisolone administered ovariectomized rats were 4.7±1.1 (p<0.001) and 11.4±2.1 (p<0.001), respectively. Methylprednisolone administered ovariectomized rats had an average osteocalcin level of 3.9±1.2 (p<0.001) (Table II). 

**Bone ALP activity**

Average alkaline phosphatase (ALP) activity level was 147.1±14 in the ovariectomized control group, 82.2±11 (p<0.0001) in the intact control group, 140.7±18 (p>0.05) in the metyrosine administered ovariectomized rat group, 107±11 (p<0.02) in the alendronate administered ovariectomized rat group, 96.7±9.7 (p<0.001) in the metyrosine and methylprednisolone administered ovariectomized rat group, 63.2±12 (p>0.0001) in the alendronate and methylprednisolone administered ovariectomized rat group, and 50.3±8 (p<0.0001) in the methylprednisolone administered ovariectomized rat group (Table II).

**Table I:** Comparison of the effects of methylprednisolone and alendronate on bone mineral density (BMD) and Bone Mineral Content (BMC) in ovariectomised and metyrosine given rats

**Drugs**	**Doses (mg/kg)**	**N**	**BMC (gr)**	**Area (cm** ^2^ **)**	**BMD (gr/cm** ^2^ **)**	**P for BMD ratio**
Methyltyrosine (ovariectomised)	20	10	0.245±0.02	1.727	0.14±0.04	0.060
Alendronate (ovariectomised)	1	10	0.258±0.03	1.803	0.143±0.01	0.045
Methyltyrosine+methylprednisolone (ovariectomised)	20+10	10	0.288±0.03	1.828	0.158±0.02	0.001
Alendronate+ methylprednisolone	1+10	10	0.238±0.02	1.76	0.132±0.03	0.336
Methylprednisolone (ovariectomised)	10	10	0.218±0.01	1.735	0.126±0.02	0.028
Intact	-	10	0.292±0.03	1.827	0.159±0.05	0.0001
Control (ovariectomised)	-	10	0.237±0.01	1.728	0.137±0.03	-

Results are mean±SEM. N: The number of rats. One-way analysis of the variance was used to evaluate the results.

P<0.05 was considered significant when compared to ovariectomised control group in LSD test.

**Table II:** Comparison of the effects of methylprednisolone and alendronate on bone osteocalsin and alkaline phosphatase (ALP) in ovariectomised and metyrosine given rats

**Drugs**	**Doses (mg/kg)**	**N**	**Osteocalsin**	**P for osteocalsin**	**ALP**	**P for ALP**
Methyltyrosine (ovariectomised)	20	10	17.1±3.5	0.075	140.7±18	0.08
Alendronate (ovariectomised)	1	10	14.3±2.7	0.01	107±11	0.02
Methyltyrosine+ methylprednisolone (ovariectomised)	20+10	10	11.4±2.1	0.001	96.7±9.7	0.001
Alendronate+ methylprednisolone	1+10	10	4.7±1.1	0.001	63.2±12	0.0001
Methylprednisolone (ovariectomised)	10	10	3.9±1.2	0.001	50.3±8	0.0001
Intact	-	10	12.1±2.7	0.001	82.2±11	0.0001
Control (ovariectomised)	-	10	20±5.3	-	147.1±14	-

Results are mean ±SEM. N: The number of rats. One-way analysis of the variance was used to evaluate the results.

P<0.05 was considered significant when compared to ovariectomised control group in LSD test. ALP: Al.

## Discussion

The occurrence of osteoporosis and BDM level osteoblast and osteoclast numbers were evaluated using bone osteocalcin and ALP estimations. Our results show that BMD levels in the ovariectomized control group are decreased compared to intact animals. The group with the lowest BMD level was the Oophorectomy +methylprednisolone group. 

In the methylprednisolone +metyrosine group +Oophorectomy, BMD levels were significant and much higher than the ovariectomized control group. When compared to the ovariectomized control group, alendronate significantly prevented the decrease of BMD in ovariectomized rats, while it did’nt do so in the alendronate +methylprednisolone +oophorectomy group. BMD estimation is used in order to prove and support osteoporosis diagnosis, to evaluate the level of osteoporosis and follow the efficiency of treatment, and to determine if treatment is necessary (15). BMD levels are decreased in estrogen deficiency (post menopause). Estrogen administration provides increase in BMD in the long term (16). This demonstrates that estrogen is associated with BMD (4). Low levels of BMD in oopherectomised rats in our study are in agreement with the literature. Glucocorticoids are shown to decrease BMD (7). 

In our study, methylprednisolone, a glucocorticoid, significantly increased BMD in metyrosine administered and ovariectomized rats. Using methyltyrosine, the amount of endogenous adrenaline can be suppressed by 30-80% (17). No data is available about the effects of the use of methyltyrosine on its own and its combined use with glucocorticoids on the bones. Data is available as to the occurrence of gastroprotective effects in animals by 50,100 and 200 mg/kg doses of methyltyrosine. Moreover, such doses of methyltyrosine protected the tissue against inflammation. It was shown that the protective effects of methyltyrosine on the tissues were occurred by inhibiting the COX-2 enzyme, without affecting the COX-1 enzyme (18). It has been reported that the prostaglandin E_2_ (PGE2), production of which is increased by COX-2 activation, resulted in the destruction of the bone tissue (19). Bezerra *et al* stated that PGE2s stimulated the osteoclasts responsible for bone destruction (20). 

While methyltyrosine given to patients with hypercalcemia causes a fall in the amount of catecholamine, it failed to prevent hypercalcemia (22). It is common knowledge that hyperparathyroidism-induced hypercalcemia is among the risk factors resulting in osteoporosis (23). In our study too, methyltyrosine failed to significantly prevent bone loss. Low levels of BMD in rats with ovariectomy might have increased by estrogen over the α-2 adrenergic receptor. This is because it has been reported that estrogen stimulates α-2 adrenergic receptors and creates a protective effect around these receptors (24).

No data is available about the direct effects of adrenalin on bone metabolism in the literature. But it was shown that sympathetic stimulation led to a decrease in BMD level. It was also reported that the negative effect of sympathetic stimulation on BMD level was antagonised by adrenergic receptor blockers (25) Alendronate was shown to increase BMD in clinical studies (26). It was shown that bone destruction is higher than bone production in osteoporosis. Therefore, osteoclast biology gains importance    (28) . In our study, osteoclast numbers in the ovariectomized control and methylprednisolone +Oophorectomy and groups were increased compared to the intact group. In the metyrosine +Oophorectomy group, osteoclast activity was lightly suppressed and osteoblast activity was stimulated. 

Oophorectomy and metyrosine, and methylprednisolone combination led to greater suppression of osteoclasts and increased stimulation of osteoblasts. Alendronate and Oophorectomy combination stimulated osteoblasts, while alendronate and Oophorectomy and methylprednisolone combination did not prevent the loss of osteoblasts. In the absence of estrogen (post-menopause term or ovariectomy) or, in long term, glucocorticoid use, a decrease in osteoblast numbers and increase in osteoclast numbers are observed (4, 5, 7). In contrast to osteoclasts, osteoblasts play a role in the synthesis, organization, and mineralization of bone matrix elements (31). 

Osteocalcin is one of the bone matrix elements produced by osteoblasts and a bone matrix-specific, non-collagenase protein. Its level in circulation indicates bone production. Osteocalcin is specific indicator of osteoblast activity. Osteoclast levels increase in the post-menopause term and return to normal levels through estrogen administration. An increase of osteocalcin in the postmenopausal period is expected to be the reaction of osteoblasts against osteoclastic activity, which leads to bone damage (a reaction of osteoblasts against osteoclastic activity (32). 

A high level of osteocalcin in the ovariectomized control group correlates with the findings in the literature. When compared to the ovariectomized control group, the low levels of osteocalcin in the alendronate administered ovariectomized group and metyrosine and methylprednisolone combination administered ovariectomized group indicates that osteoblastic reaction was not seriously triggered due to the absence of osteoclasts in these groups. 

A much lower level of osteocalcin in the methylprednisolone administered ovariectomized group suggests strong suppression of osteoblasts by methylprednisolone. The osteocalcin level was also low in the alendronate and methylprednisolone administered ovariectomized group. This shows that alendronate was not able to prevent severe bone damage in the methylprednisolone administered ovariectomized rats because the most severe osteoporosis was found in methylprednisolone administered ovariectomized rat group. 

This may arise from a methylprednisolone-induced decrease in the number of osteoblasts and suppression of osteoblastic activity against osteoclasts. It is known that number of osteoclasts increase and number of osteoblasts is not affected by ovariectomy. Glucocorticoids decrease osteocalcin levels. Osteocalcin is a glycoprotein, highly sensitive to the inhibitory effect of glucocorticoids (33).

In the alendronate+ Oophorectomy and metyrosine+ Oophorectomy groups, osteocalcin levels were lower than the ovariectomized control group, as expected, either because there were no osteoclasts (alendronate) or too few osteoclasts to start osteoblast reaction (metyrosine). Much lower osteocalcin levels in the methylprednisolone+ Oophorectomy group may be due to serious suppression of osteoblasts, the source of osteocalcin. Methylprednisolone suppressed osteoblast reaction in the alendronate+ Oophorectomy group, while it did not do so in the metyrosine+ Oophorectomy group. 

Another means of osteoblastic activity estimation is the bone ALP estimation. ALP is an early indicator of bone production, while osteocalcin is a late indicator. An increase in bone loss results in an increase in ALP. ALP is the most reliable indicator of bone production (34). Because ALP is a product of osteoblasts, like osteocalcin, they are both response reactions of osteoblasts. Therefore, serum ALP levels may double in the post-menopause term (ovariectomy), dependent of the increase in bone cycle (35). A decrease in ALP levels after corticosteroid treatment (36) supports our results. 

## Conclusion

In conclusion, osteoporosis in ovariectomized rats intensified with methylprednisolone. Alendronate only prevented Oophorectomy related osteoporosis, but it did not prevent severe osteoporosis in the methylprednisolone+ Oophorectomy group. Methylprednisolone was the drug that most effectively prevented osteoporosis in ovariectomized+ metyrosine administered rats. 
